# Salutatory

**Published:** 1881-01

**Authors:** 


### HOMŒOPATHIC PHYSICIAN.

A MONTHLY JOURNAL OF MEDICAL SCIENCE.

"If our school ever gives up the strict inductive method of Hahnemann, we are lost, and deserve to be mentioned only as a caricature, in the history of medicine." — CONSTANTINE HERING.

Vol. I. JANUARY, 1881. No. 1.

### SALUTATORY.

CONSTANTINE HERING, whom we may justly call the American Hahnemann, wrote, just before his death, those warning words which appear at the top of our page. This admonition we shall keep displayed at our mast-head, to serve as a beacon light of warning to the foolhardy practitioner, who would desert our law, the true and unerring compass of therapeutics, and trust to chance knowledge of the rocky coast, or hidden sand-bars which the practitioner continually meets in his stormy cruise against disease and death.

Soon after the demise of our late teacher and guide, some friends of the departed hero, and earnest workers for pure Homœopathy, met to consider how his last injunction could be best obeyed, and how the life work of this prince of workers should not be rendered nugatory, nor our noble school live "as a caricature, in the history of medicine."

To assist in this noble work a pure, able Homœopathic journal was considered necessary. And, that this journal might exert a powerful influence for good, in our school, it was determined to ask the active co-operation of the best and ablest men in our ranks. This was done; and the hearty, willing offers of assistance which came back to us were very gratifying, and moreover assured us of success; it was even more pleasing than this, as it proved our best men were alive to the danger, and eager to meet it.

As to our course and work, we may say, THE HOMŒOPATHIC PHYSICIAN—so called because the Homœopathic physician represents the full complement of scientific medicine, and because Hahnemann considered it a title of the highest honor—will strive to show that the conscientious practitioner preserves intact "the strict inductive method of Hahnemann," also that the following are the true and essential features of Homœopathy:

The Law of the Similars.
The Single Remedy.
The Minimum Dose.

The first being the unfailing law; the last two its logical corallaries.

To establish these principles, THE HOMŒOPATHIC PHYSICIAN will offer logical argument and clinical proof; all "fatal errors," made by those attempting to pervert these principles, all deviations from the strict application of *the Law*, will be courteously, yet *fearlessly* combated. In short, this new advocate for professional favor will defend unflinchingly in its pages that law which has never failed its editors in the sick room.

A large portion of its work will be clinical matter furnished by our able corps of distinguished contributors; the Materia Medica will be fully conpared, corrected and illustrated by the best therapeutists in the Homœopathic School; current medical literature will be thoroughly scanned for interesting or instructive matter; books will be impartially reviewed; the papers will aim to be short, clear and to the point.

The law of similars is to Homœopathy what the "vital spark" is to the human frame; crush it out and we are but a dead mass, certain to become corrupt and decayed. To preserve this law, this vital spark, should be the earnest desire of all true men and all earnest physicians. No true man could oppose such a noble work — noble, for it seeks to preserve and to perfect a science whose sole object is to relieve and cure human misery. To this work is this journal dedicated and for this purpose is it established. We ask the aid of all true Homœopaths, promising to be "independent in every thing, neutral in nothing."

